# Association of physiological factors with grip and leg extension strength: tohoku medical megabank community-based cohort study

**DOI:** 10.1186/s12889-024-18244-z

**Published:** 2024-03-05

**Authors:** Yoshiaki Noji, Rieko Hatanaka, Naoki Nakaya, Mana Kogure, Kumi Nakaya, Ippei Chiba, Ikumi Kanno, Tomohiro Nakamura, Naho Tsuchiya, Haruki Momma, Yohei Hamanaka, Masatsugu Orui, Tomoko Kobayashi, Akira Uruno, Eiichi N Kodama, Ryoichi Nagatomi, Nobuo Fuse, Shinichi Kuriyama, Atsushi Hozawa

**Affiliations:** 1 Graduate School of Medicine, Tohoku University, 2-1, Seiryo-machi, Aoba-ku, 980-8574 Sendai, Miyagi Japan; 2 Department of Rehabilitation, Faculty of Health Science, Tohoku Fukushi University, Aoba-ku, Sendai, Miyagi Japan; 3 Tohoku Medical Megabank Organization, Tohoku University, Aoba-ku, Sendai, Miyagi Japan; 4 Faculty of Data Science, Kyoto Women’s University, Higashiyama-ku, Kyoto, Japan; 5 Tohoku University Hospital, Tohoku University, Aoba-ku, Sendai, Miyagi Japan; 6 International Research Institute of Disaster Science, Tohoku University, Aoba-ku, Sendai, Miyagi Japan

**Keywords:** General population, Grip strength, Leg extension strength, Muscle strength, Physiological data

## Abstract

**Background:**

Upper and lower extremity muscle strength can be used to predict health outcomes. However, the difference between the relation of upper extremity muscle and of lower extremity muscle with physiological factors is unclear. This study aimed to evaluate the association between physiological data and muscle strength, measured using grip and leg extension strength, among Japanese adults.

**Methods:**

We conducted a cross-sectional study of 2,861 men and 6,717 women aged ≥ 20 years living in Miyagi Prefecture, Japan. Grip strength was measured using a dynamometer. Leg extension strength was measured using a hydraulic isokinetic leg press machine. Anthropometry and physiological data, including blood pressure, calcaneal ultrasound bone status, pulmonary function, carotid echography, and blood information, were assessed. We used a general linear model adjusted for age, body composition, and smoking status to evaluate the association between muscle strength and physiological factors.

**Results:**

Grip and leg extension strength were positively associated with bone area ratio, vital capacity, forced vital capacity, forced expiratory volume in one second, and estimated glomerular filtration rate, and negatively associated with waist circumference and percentage body fat mass in both the sexes. Diastolic blood pressure was positively associated with grip strength in both the sexes and leg extension strength in men, but not women. High-density lipoprotein cholesterol and red blood cell counts were positively associated with grip and leg extension strength in women, but not men. In both the sexes, pulse rate, total cholesterol, and uric acid were consistently associated with only leg extension strength, but not grip strength. In women, glycated hemoglobin demonstrated negative and positive associations with grip and leg extension strength, respectively.

**Conclusions:**

Grip and leg extension strength demonstrated similar associations with anthropometry, pulmonary function, and estimated glomerular filtration rate, but the associations with the other factors were not always consistent.

**Supplementary Information:**

The online version contains supplementary material available at 10.1186/s12889-024-18244-z.

## Background

Low muscle strength is associated with successful aging [1], mortality [2–7],and diseases, such as cancer [2, 3], cardiovascular disease [2, 3], respiratory disease [2]. However, the reason for this association remains unclear. The causal association between low muscle strength and health outcomes necessitates the investigation of the underlying factors. Moreover, a meta-analysis (comprising 38 studies with 1,907,580 participants) published in 2018 indicated that upper and lower limb muscular strength was associated with the risk of mortality in adults, regardless of age or follow-up period [8]. Moreover, Sanderson et al. reported that combined measures of grip strength and lower limb indicators better predict survival than grip strength alone [5]. Therefore, mechanisms affecting the association between low muscle strength and health outcomes might differ between the upper and lower extremity muscle strength.

Studies have reported on associations between muscle strength and physiological parameters in large populations [9–17]. A large population-based cross-sectional study in Switzerland showed an association between muscle strength and cardiovascular risk markers such as waist circumference (WC) and fat mass [9]. Moreover, muscle strength was associated with cardiovascular risk factors, including blood pressure [10, 13], diabetes [10, 11, 16], lipid makers [10, 13], and atherosclerosis [14, 15]. Furthermore, besides cardiovascular risk factors, muscle strength is also associated with anemia [12] and pulmonary function [17].

However, most studies have only emphasized grip strength as a measure of muscle strength. Furthermore, the association between physiological data and muscle strength, as measured using grip and leg extension strength, has not been widely investigated in the general population. To investigate complementary mechanisms underlying the association between muscle strength and health outcomes, it is necessary to clarify the differences in the associations among upper and lower extremity muscle strength and physiological data. Therefore, clarifying the significance of each muscle strength may indicate mortality and disease development and help decide appropriate interventions.

Thus, this study aimed to assess the association between physiological data and muscle strength, measured using grip and leg extension strength, in a large community-based sample aged 20–89 years.

## Methods

### Participants

This cross-sectional study used data from the Tohoku Medical Megabank Community-Based Cohort Study, a population-based prospective cohort study of individuals aged ≥ 20 years in Miyagi and Iwate Prefectures, Japan [18–36]. Details of recruitment have been described previously [18]. Participants underwent several physiological measurements, such as grip and leg extension strength. Moreover, we considered it appropriate to use blood data collected at the same time. Therefore, we used participants from a type 2 survey performed in Miyagi.

**Fig. 1:**
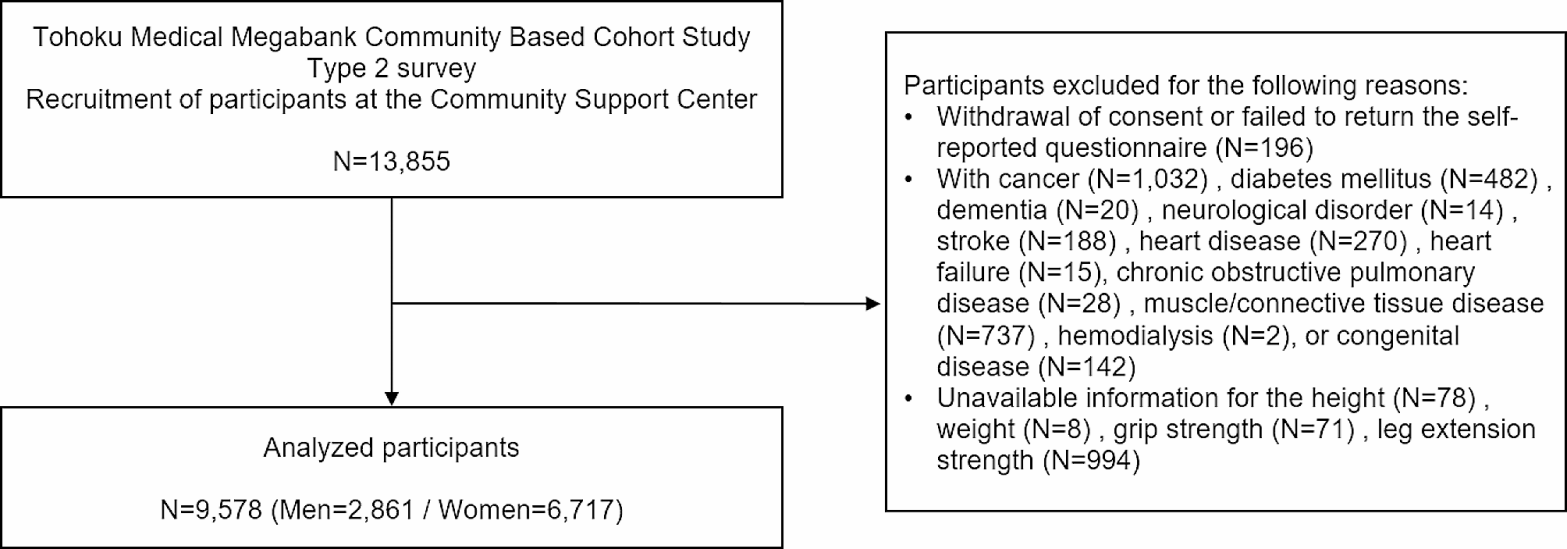
Flowchart of participant selection

We excluded 13,855 participants in the type 2 survey who withdrew consent or failed to return the self-reported questionnaire (*N* = 196). Considering the impact of the association between muscle strength and physiological data, participants with a self-reported history of cancer (*N* = 1,032), diabetes mellitus (*N* = 482), dementia (*N* = 20), neurological disorder (*N* = 14), stroke (*N* = 188), heart disease (*N* = 270), heart failure (*N* = 15), chronic obstructive pulmonary disease (*N* = 28), muscle/connective tissue disease (*N* = 737), hemodialysis (*N* = 2), or congenital disease (*N* = 142) were excluded. Furthermore, participants without information on height (*N* = 78), weight (*N* = 8), grip strength (*N* = 71), or leg extension strength (*N* = 994) were excluded. Finally, data from 9,578 participants were analyzed (Fig. 1).

### Muscle strength measurement

Grip strength was measured using a dynamometer (YD 110 kg; Tsutsumi Co., Ltd., Tokyo, Japan). The participants were instructed to stand with their arms down along their body and hold the dynamometer. Then, they squeezed the handle of the dynamometer as hard as possible. The participants performed the test twice for each hand alternately, and the highest value was used [6, 14]. We measured leg extension strength using a hydraulic isokinetic leg-press machine (T.K.K.1865; Takei Scientific Instruments Co., Ltd., Niigata, Japan). The participants sat on the seat of the leg-press machine in a 90° knee joint position and had belts fixed on their legs and waist. Subsequently, we instructed them to grip the bars and kick as fast as possible from the 90° knee joint position while keeping their legs extended. More than one measurement (up to five) was performed, and the maximum value was used for the analysis.

### Other data

We assessed several physiological data such as cardiovascular risk factors, pulmonary function, and renal function. Alcohol drinking, smoking, and educational status were assessed using a self-administered questionnaire. Alcohol drinking status was classified as “never-drinker” (those who selected “little or no drinking” or “constitutionally unable to drink”), “ex-drinker (those who selected “no longer”), “current-drinker” (those who selected “drinking”), or “unknown” (those who did not respond to the question). Smoking status was classified as “never-smoker” (those who smoked < 100 cigarettes in their lifetime), “ex-smoker” (those who smoked ≥ 100 cigarettes in their lifetime, but answered “current non-smoker” in the questionnaire), “current-smoker” (those who smoked ≥ 100 cigarettes in their lifetime and answered “current smoker” in the questionnaire), or “unknown” (those who did not answer the question). Education status was classified as “<12 years” (those who selected “elementary school or junior high school”), “12 years” (those who selected “high school”), “>12 years” (those who selected “vocational school,” “junior college or technical college,” “university,” or “graduate school”), “others” (those who selected “other”), or “unknown” (those who did not answer the question).

Participant height was measured to the nearest 0.1 cm using a stadiometer (AD-6400; A&D Co., Ltd.). Weight and percentage of body fat mass were measured using a body composition analyzer (InBody720; Biospace Co., Ltd., Seoul, Korea). The weight was measured in increments of 0.1 kg, and 1.0 kg was subtracted to account for the weight of the participant’s clothing. Body mass index (BMI) was calculated as the weight divided by the height squared. WC was measured at the level of the umbilicus using a tape measure.

Blood pressure and pulse rate were measured twice in the right upper arm using a digital automatic blood pressure monitor (HEM-9000AI; Omron Healthcare Co., Ltd., Kyoto, Japan) after the participants rested in a sitting position for ≥ 2 min, and the average value was used. The number of steps per day was counted using a pedometer (HJ-205IT; Omron Healthcare Co., Ltd., Kyoto, Japan). Participants were instructed to wear a pedometer throughout the day for 2 weeks, except while bathing and sleeping. Bone area ratio was assessed only once in the sitting position using an ultrasound bone densitometer (UBM-3000; Ishikawa Seisakusho, Ltd., Ishikawa, Japan) on the right calcaneus and was used as an indicator of the bone status [37, 38]. Pulmonary functions, such as vital capacity (VC), forced vital capacity (FVC), forced expiratory volume in 1 s (FEV1), and forced expiratory volume in 1 s as a percentage of the forced vital capacity (FEV1/FVC), were measured in the sitting position using a spirometer (HI-801; Chest M.I., Inc., Tokyo, Japan). Carotid intima-media thickness (CIMT) was measured using ultrasonography (GM-72P00A; Panasonic Healthcare Co., Ltd., Tokyo, Japan) and calculated as the CIMT measurement with the carotid plaque excluded. We used the mean right CIMT values for the analysis employing a software program that provides the mean thickness of the IMT complex on the common carotid artery [39].

Blood samples were collected under non-fasting conditions. The details of the measurement methods have been described previously [18]. We estimated the glomerular filtration rate (eGFR) from the serum cystatin C levels using the following equation developed for the Japanese population [40, 41]:

$${\rm{eGFR}}\,\left( {{\rm{mL}}\,{\rm{/}}\,{\rm{min}}\,{\rm{/}}\,{\rm{1}}{\rm{.73}}\,{{\rm{m}}^{\rm{2}}}} \right)$$

$$\eqalign{& \left\{ {{\rm{104}}\,{\rm{ \times }}\,{{\left[ {{\rm{serum}}\,{\rm{cystatin}}\,{\rm{C}}\,\left( {{\rm{mg}}\,{\rm{/}}\,{\rm{L}}} \right)} \right]}^{{\rm{(}} - {\rm{1}}{\rm{.019)}}}}\,{\rm{ \times }}\,{\rm{ 0}}{\rm{.99}}{{\rm{6}}^{{\rm{[age (years)]}}}}} \right\} \cr & \, - \,{\rm{8}}\,\left( {{\rm{for}}\,{\rm{men}}} \right){\rm{,}} \cr & \left\{ {{\rm{104}}\,{\rm{ \times }}\,{{\left[ {{\rm{serum}}\,{\rm{cystatin}}\,{\rm{C}}\,\left( {{\rm{mg}}\,{\rm{/}}\,{\rm{L}}} \right)} \right]}^{{\rm{(}} - {\rm{1}}{\rm{.019)}}}}\,{\rm{ \times }}\,{\rm{0}}{\rm{.99}}{{\rm{6}}^{{\rm{[age (years)]}}}}\,{\rm{ \times }}\,{\rm{0}}{\rm{.929}}} \right\} \cr & \, - \,{\rm{8}}\,\left( {{\rm{for}}\,{\rm{women}}} \right). \cr} $$

### Statistical analysis

Grip and leg extension strength were divided into quartiles, and participant characteristics were compared using analysis of variance (ANOVA) for continuous variables and chi-square test for categorical variables. We used a general linear model to evaluate whether the muscle strength, as measured using the grip or leg extension strength, was associated with the physiological data. The results are expressed as multivariate-adjusted coefficients. General linear models were adjusted for age (continuous), smoking status (never smoker/ex-smoker/current smoker/unknown), and BMI. Weight (continuous) was used as an adjustment factor instead of the BMI [9] when using WC as the explanatory variable. Similarly, considering that pulmonary function is affected by age and height [42], the height (continuous) was used as an adjustment factor instead of the BMI, when using the VC, FVC, FEV1, and FEV1/FVC as the explanatory variables. We also performed subgroup analysis according to age (< 65, 65–74, and > 74 years).

The associations between grip strength and physiological data and leg strength and physiological data were consistent when the coefficients for the grip and leg extension strength were significantly positive or negative. A statistically significant association with grip or leg extension strength, but not with the other factors, was defined as a different association. Furthermore, if one of the regression coefficients for grip and leg extension strength was significantly positive and the other was significantly negative, the associations between the grip or leg extension strength and physiological data were different. Statistical significance was set at *p* < 0.05. All analyses were performed using the Statistical Analysis System software, version 9.4 for Windows (SAS Inc., Cary, NC, USA).

## Results

**Fig. 2:**
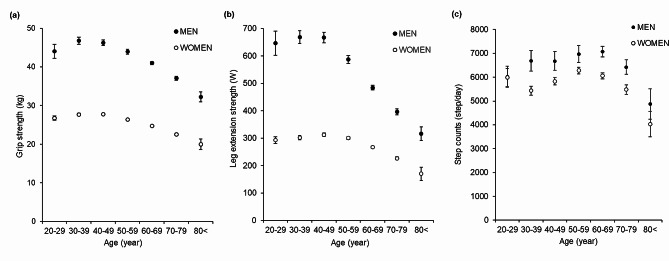
Age-dependent changes in the (**a**) grip strength, (**b**) leg extension strength and (**c**) step count. Error bar plot of grip and leg extension strength by sex. Error bars represent 95% confidence intervals for the means

Figure 2 depicts the age-dependent changes in muscle strength measured using grip or leg extension strength. Age was inversely associated with grip and leg extension strength. Men had higher grip and leg extension strength than women; however, the difference decreased after 50 years of age.

### Association between grip strength and physiological data

Table 1 summarizes the participant characteristics according to the grip strength quartiles. In men, physiological data, excluding pulse rate, showed differences between the grip strength quartile groups. In women, physiological data, excluding diastolic blood pressure and high-density lipoprotein (HDL) cholesterol, showed differences between the grip strength quartile groups.

**Table 1:** Characteristics of the participants according to the quartile of relative grip strength

	Quartile of grip strength (MEN)					Quartile of grip strength (WOMEN)				
	Q1 < 37 kg	Q2 37–41 kg	Q3 42–47 kg	Q4 > 47 kg	p-value	Q1 < 23 kg	Q2 23–26 kg	Q3 27–29 kg	Q4 > 29 kg	p-value
Age, years	64.2 (14.0)	60.1 (13.4)	55.5 (13.1)	48.9 (13.0)	< 0.0001	59.0 (13.3)	54.7 (13.2)	51.7 (12.9)	47.3 (12.9)	< 0.0001
LES, W	411.3 (149.9)	491.9 (153.2)	569.2 (163.2)	677.9 (201.1)	< 0.0001	241.3 (84.3)	274.9 (87.9)	302.7 (96.2)	339.6 (109.5)	< 0.0001
Alcohol drinking status, n (%)					0.5614					< 0.0001
Current-drinker	649 (77.5)	481 (80.3)	604 (80.2)	544 (81.0)		922 (44.1)	835 (48.4)	727 (49.6)	760 (53.0)	
Ex-drinker	30 (3.6)	16 (2.7)	16 (2.1)	16 (2.4)		31 (1.5)	21 (1.2)	33 (2.3)	19 (1.3)	
Never-drinker	157 (18.8)	102 (17.0)	133 (17.7)	112 (16.7)		1138(54.4)	868 (50.3)	706 (48.2)	653 (45.6)	
Unknown	1 (0.1)	0 (0.0)	0 (0.0)	0 (0.0)		2 (0.1)	1 (0.1)	0 (0.0)	1 (0.1)	
Smoking status, n (%)					< 0.0001					< 0.0001
Current-smoker	158 (18.9)	110 (18.4)	176 (23.4)	231 (34.4)		150 (7.2)	111 (6.4)	146 (10.0)	148 (10.3)	
Ex-smoker	387 (46.2)	323 (53.9)	383 (50.9)	266 (39.6)		246 (11.8)	225 (13.0)	204 (13.9)	282 (19.7)	
Never-smoker	290 (34.6)	166 (27.7)	193 (25.6)	174 (25.9)		1691(80.8)	1387(80.4)	1112(75.9)	1001 (69.9)	
Unknown	2 (0.2)	0 (0.0)	1 (0.1)	1 (0.1)		6 (0.3)	2 (0.1)	4 (0.3)	2 (0.1)	
Education status, n (%)					0.0002					< 0.0001
<12 years	94 (11.2)	45 (7.5)	55 (7.3)	45 (6.7)		155 (7.4)	83 (4.8)	53 (3.6)	32 (2.2)	
12 years	396 (47.3)	243 (40.6)	343 (45.6)	344 (51.2)		1084 (51.8)	874 (50.7)	669 (45.6)	638 (44.5)	
>12 years	332 (39.7)	297 (49.6)	349 (46.3)	276 (41.1)		837 (40.0)	757 (43.9)	731 (49.9)	750 (52.3)	
Others	6 (0.7)	5 (0.8)	3 (0.4)	2 (0.3)		6 (0.3)	4 (0.2)	5 (0.3)	4 (0.3)	
Unknown	9 (1.1)	9 (1.5)	3 (0.4)	5 (0.7)		11 (0.5)	7 (0.4)	8 (0.5)	9 (0.6)	
Anthropometry										
Height, cm	164.7 (6.0)	167.4 (5.6)	169.2 (5.9)	171.9 (5.6)	< 0.0001	153.3 (5.4)	156.0 (5.1)	157.4 (5.0)	159.8 (5.1)	< 0.0001
Weight, kg	62.7 (9.0)	65.2 (8.5)	68.4 (9.5)	73.5 (11.5)	< 0.0001	51.2 (7.7)	53.6 (7.8)	54.8 (8.4)	58.0 (9.9)	< 0.0001
BMI, kg/cm^2^	23.1 (2.9)	23.3 (2.8)	23.9 (3)	24.9 (3.6)	< 0.0001	21.8 (3.2)	22.0 (3.2)	22.1 (3.4)	22.7 (3.8)	< 0.0001
WC, cm	84.9 (8.3)	85.1 (7.9)	86.1 (8.4)	88.2 (9.6)	< 0.0001	80.6 (9.2)	81.1 (9.1)	80.6 (9.3)	82.0 (10.1)	< 0.0001
%BFM, %	24.7 (6.2)	23.1 (5.8)	22.9 (6.2)	22.4 (6.4)	< 0.0001	31.2 (7.0)	30.5 (7.0)	29.5 (7.0)	29.2 (7.5)	< 0.0001
Bone area ratio, %	28.0 (3.8)	28.3 (3.6)	28.9 (3.6)	29.3 (3.7)	< 0.0001	27.4 (4.0)	28.2 (4.1)	28.7 (4.2)	29.8 (4.4)	< 0.0001
Pulmonary function										
VC, L	3.7 (0.6)	4.0 (0.6)	4.2 (0.6)	4.5 (0.7)	< 0.0001	2.7 (0.4)	2.9 (0.4)	3.0 (0.4)	3.3 (0.5)	< 0.0001
FVC, L	3.5 (0.7)	3.8 (0.6)	4.0 (0.6)	4.4 (0.7)	< 0.0001	2.6 (0.5)	2.8 (0.5)	3.0 (0.4)	3.2 (0.5)	< 0.0001
FEV1, L	2.8 (0.6)	3.0 (0.6)	3.2 (0.6)	3.5 (0.6)	< 0.0001	2.1 (0.4)	2.3 (0.4)	2.4 (0.4)	2.6 (0.4)	< 0.0001
FEV1/FVC, %	78.4 (7.5)	79.2 (7.3)	79.3 (6.4)	81 (5.7)	< 0.0001	81.1 (6.0)	82.6 (5.8)	81.9 (5.8)	82.6 (5.7)	< 0.0001
Blood pressure										
SBP, mmHg	137.2 (17.7)	134.7 (17.7)	133.9 (16.7)	132.2 (15.3)	< 0.0001	127.6 (18.8)	126.4 (18.8)	124.2 (18.1)	122.7 (17.4)	< 0.0001
DBP, mmHg	80.6 (10.8)	81.8 (11.2)	82.9 (10.8)	83.2 (11.3)	< 0.0001	76.8 (10.5)	77.2 (11.0)	76.4 (10.6)	77.0 (11.1)	0.2382
Pulse rate, beat/min	65.3 (11.3)	64.7 (10.2)	64.6 (9.8)	65.8 (9.8)	0.1357	67.0 (9.5)	66.2 (9.1)	66.7 (9.4)	67.2 (9.8)	0.0092
CIMT, mm	0.7 (0.2)	0.6 (0.1)	0.6 (0.1)	0.6 (0.1)	< 0.0001	0.6 (0.1)	0.6 (0.1)	0.6 (0.1)	0.5 (0.1)	< 0.0001
Lipid markers										
TC, mg/dL	201.6 (33.3)	203.0 (33.6)	206.7 (37.6)	203.2 (34.4)	0.0065	215.0 (34.5)	215.0 (37.1)	211.4 (36.4)	205.9 (35.3)	< 0.0001
Triglycerides, mg/dL	114.1 (72.1)	123.2 (100.5)	128.8 (126.8)	140.8 (105.5)	< 0.0001	96.0 (58.5)	92.0 (72.1)	92.6 (71.1)	86.7 (56.4)	0.0005
HDL-C, mg/dL	58.0 (15.6)	57.9 (14.5)	57.6 (15.2)	55.1 (14.1)	0.0005	67.6 (15.8)	68.5 (15.7)	67.8 (16.4)	67.5 (16.6)	0.3437
Glucometabolic markers										
HbA1c, %	5.6 (0.5)	5.5 (0.5)	5.5 (0.4)	5.4 (0.5)	< 0.0001	5.5 (0.4)	5.4 (0.4)	5.4 (0.4)	5.4 (0.4)	< 0.0001
Glucose, mg/dL	91.8 (17.6)	90.5 (13.9)	89.2 (15.6)	89.3 (14.7)	0.0030	86.7 (12.9)	85.5 (11.7)	85.0 (12.2)	84.6 (11.1)	< 0.0001
eGFR, ml/min/1.73 m^2^	91.4 (21.7)	97.4 (19.2)	102.9 (19.7)	108.5 (19.8)	< 0.0001	102.2 (21.8)	107.1 (22.6)	111.3 (22.8)	115.9 (22.5)	< 0.0001
Uric acid, mg/dL	5.8 (1.2)	5.9 (1.2)	5.9 (1.2)	6.2 (1.3)	< 0.0001	4.5 (1.0)	4.5 (1.0)	4.4 (1.0)	4.4 (1.0)	0.0552
Red blood cells count, 10^4^/µL	470.8 (42.1)	474.5 (40.8)	482.1 (38.7)	491.8 (36.1)	< 0.0001	439.8 (32.6)	442.8 (31.9)	441.6 (32.6)	445.2 (32.1)	< 0.0001
Hemoglobin, g/dL	14.8 (1.3)	14.9 (1.1)	15.0 (1.1)	15.3 (1.0)	< 0.0001	13.3 (1.1)	13.3 (1.0)	13.2 (1.1)	13.2 (1.2)	0.0017
Hematocrit, %	44.4 (3.4)	44.7 (3.1)	45.1 (3.0)	45.7 (2.7)	< 0.0001	40.7 (2.9)	40.8 (2.7)	40.5 (3.0)	40.5 (3.0)	0.0028

Data are expressed as mean (standard deviation) for continuous variables and number (percentage) for categorical variables

LES: leg extension strength; BMI: body mass index; WC: waist circumference; %BFM; percentage body fat mass VC: vital capacity; FVC: forced vital capacity; FEV1: forced expiratory volume in 1 s; SBP: systolic blood pressure; DBP: diastolic blood pressure; TC: total cholesterol; CIMT: carotid intima-media thickness; HDL-C: high-density lipoprotein cholesterol; HbA1c: hemoglobin A1c; eGFR: estimated glomerular filtration rate; and Q: quartile

The p-value was obtained using ANOVA for continuous variables and chi-square for categorical variables of proportion

After adjusting for age, body composition, and smoking status, grip strength was positively associated with bone area ratio, VC, FVC, FEV1, diastolic blood pressure, and eGFR, and negatively associated with WC, percentage body fat mass, and hemoglobinA1c (HbA1c) in both the sexes (Table 2). In men, grip strength was negatively associated with CIMT. In women, it was positively associated with systolic blood pressure, high-density lipoprotein cholesterol levels, and red blood cell count.

**Table 2:** Association between grip strength and physiological data adjusted for age, body composition, and smoking

	Men			Women		
	N	β	p-value	N	β	p-value
Anthropometry						
WC^a^, cm	2855	–0.407	< 0.0001	6701	–0.157	< 0.0001
%BFM, %	2851	–0.770	< 0.0001	6704	–0.391	< 0.0001
Bone area ratio, %	2858	0.121	0.0007	6713	0.092	< 0.0001
Pulmonary function						
VC^b^, L	2716	2.668	< 0.0001	6375	2.535	< 0.0001
FVC^b^, L	2708	2.345	< 0.0001	6350	2.513	< 0.0001
FEV1^b^, L	2708	2.665	< 0.0001	6350	2.705	< 0.0001
FEV1/FVC^b^, %	2708	0.033	0.1078	6350	0.000	0.9851
Blood pressure						
SBP, mmHg	2857	0.011	0.1738	6705	0.013	0.0001
DBP, mmHg	2857	0.053	< 0.0001	6705	0.019	0.0003
Pulse rate, beat/min	2857	–0.023	0.0666	6705	–0.005	0.4072
CIMT, mm	2845	–2.401	0.0216	6677	0.655	0.2269
Lipid markers						
TC, mg/dL	2858	0.004	0.3362	6710	0.003	0.0832
Triglycerides, mg/dL	2858	0.001	0.4133	6710	–0.002	0.0545
HDL-C, mg/dL	2858	0.015	0.0911	6710	0.009	0.0123
Glucometabolic markers						
HbA1c, %	2856	–0.730	0.0099	6701	–0.385	0.0095
Glucose, mg/dL	2856	–0.004	0.6317	6701	–0.001	0.8268
eGFR, ml/min/1.73 m^2^	2858	0.044	< 0.0001	6710	0.009	0.0085
Uric acid, mg/dL	2858	0.118	0.2730	6709	–0.054	0.3447
Red blood cell count, 10^4^/µL	2858	0.001	0.7214	6708	0.004	0.0224
Hemoglobin, g/dL	2858	0.078	0.5088	6708	0.052	0.2927
Hematocrit, %	2858	0.046	0.2957	6708	0.030	0.1103

The general linear model was adjusted for age, smoking status, and body mass index

^a^Adjusted for weight instead of body mass index

^b^Adjusted for height instead of body mass index

WC: waist circumference; %BFM: percentage body fat mass; VC: vital capacity; FVC: forced vital capacity; FEV1: forced expiratory volume in 1 s; SBP: systolic blood pressure; DBP: diastolic blood pressure; CIMT: carotid intima-media thickness; TC: total cholesterol; HDL-C: high-density lipoprotein cholesterol; HbA1c: hemoglobin A1c; and eGFR: estimated glomerular filtration rate

### Association between leg extension strength and physiological data

Table 3 summarizes the participant characteristics according to the quartiles of the leg extension strength. In men, physiological data, excluding WC and HDL cholesterol, showed differences between the leg extension strength quartile groups. In women, physiological data, excluding diastolic blood pressure, uric acid, hemoglobin, and hematocrit, showed differences between the leg extension strength quartiles groups.

**Table 3:** Characteristics of the participants according to the quartile of relative leg extension strength

	Quartile of leg extension strength (MEN)					Quartile of leg extension strength (WOMEN)				
	Q1 < 393 W	Q2 394–509 W	Q3 510–639 W	Q4 > 640 W	p-value	Q1 < 214 W	Q2 215–276 W	Q3 277–347 W	Q4 > 348 W	p-value
Age, years	65.7 (12.9)	61.4 (12.5)	55.9 (13.1)	46.6 (12.4)	< 0.0001	56.6 (14.8)	55.5 (13.4)	53.8 (12.7)	49.3 (12.2)	< 0.0001
GS, kg	36.7 (6.7)	40.6 (6.5)	43.2 (6.2)	47.6 (7.5)	< 0.0001	23.8 (4.3)	25.2 (4.3)	26.1 (4.2)	28.3 (4.5)	< 0.0001
Alcohol drinking status, n (%)					0.0036					< 0.0001
Current-drinker	540 (75.4)	570 (79.4)	582 (81.4)	586 (82.3)		719 (42.5)	771 (45.5)	834 (50.2)	920 (55.0)	
Ex-drinker	32 (4.5)	16 (2.2)	21 (2.9)	9 (1.3)		27 (1.6)	24 (1.4)	21 (1.3)	32 (1.9)	
Never-drinker	143 (20.0)	132 (18.4)	112 (15.7)	117 (16.4)		944 (55.8)	896 (52.9)	805 (48.5)	720 (43.1)	
Unknown	1 (0.1)	0 (0)	0 (0)	0 (0)		1 (0.1)	2 (0.1)	1 (0.1)	0 (0)	
Smoking status, n (%)					< 0.0001					< 0.0001
Current-smoker	128 (17.9)	147 (20.5)	177 (24.8)	223 (31.3)		139 (8.2)	123 (7.3)	127 (7.6)	166 (9.9)	
Ex-smoker	349 (48.7)	358 (49.9)	364 (50.9)	288 (40.4)		184 (10.9)	212 (12.5)	262 (15.8)	299 (17.9)	
Never-smoker	237 (33.1)	211 (29.4)	174 (24.3)	201 (28.2)		1364 (80.7)	1355 (80.0)	1270 (76.5)	1202 (71.9)	
Unknown	2 (0.3)	2 (0.3)	0 (0)	0 (0)		4 (0.2)	3 (0.2)	2 (0.1)	5 (0.3)	
Education status, n (%)					< 0.0001					< 0.0001
<12 years	111 (15.5)	54 (7.5)	45 (6.3)	29 (4.1)		150 (8.9)	83 (4.9)	57 (3.4)	33 (2)	
12 years	362 (50.6)	354 (49.3)	321 (44.9)	289 (40.6)		867 (51.3)	846 (50.0)	787 (47.4)	765 (45.8)	
>12 years	229 (32.0)	299 (41.6)	335 (46.9)	391 (54.9)		653 (38.6)	751 (44.4)	809 (48.7)	862 (51.6)	
Others	5 (0.7)	3 (0.4)	7 (1.0)	1 (0.1)		7 (0.4)	2 (0.1)	5 (0.3)	5 (0.3)	
Unknown	9 (1.3)	8 (1.1)	7 (1.0)	2 (0.3)		14 (0.8)	11 (0.6)	3 (0.2)	7 (0.4)	
Anthropometry										
Height, cm	164.8 (6)	166.8 (5.8)	168.8 (5.7)	172.1 (5.6)	< 0.0001	154.2 (5.8)	155.6 (5.4)	156.6 (5.3)	158.6 (5.3)	< 0.0001
Weight, kg	63.6 (10.5)	65.6 (9.8)	67.8 (9.4)	72.1 (10.4)	< 0.0001	53.0 (9.0)	53.5 (8.7)	53.8 (8.4)	55.9 (8.5)	< 0.0001
BMI, kg/cm^2^	23.4 (3.4)	23.5 (3.0)	23.8 (3.0)	24.3 (3.1)	< 0.0001	22.3 (3.6)	22.1 (3.4)	21.9 (3.3)	22.2 (3.2)	0.0018
WC, cm	85.6 (9.3)	85.6 (8.3)	86.1 (8.2)	86.7 (8.7)	0.0660	81.4 (9.8)	81.1 (9.3)	80.6 (9.3)	80.9 (9.1)	0.0297
%BFM, %	24.9 (6.4)	23.4 (6.0)	23.2 (6.1)	21.9 (6.1)	< 0.0001	31.2 (7.4)	30.6 (7.1)	29.8 (7.1)	29.3 (6.9)	< 0.0001
Bone area ratio, %	27.7 (3.6)	28.2 (3.7)	28.7 (3.7)	29.7 (3.9)	< 0.0001	27.8 (4.2)	28.0 (4.2)	28.4 (4.1)	29.4 (4.3)	< 0.0001
Pulmonary function										
VC, L	3.6 (0.6)	3.9 (0.6)	4.1 (0.6)	4.6 (0.6)	< 0.0001	2.7 (0.5)	2.9 (0.5)	3.0 (0.5)	3.2 (0.5)	< 0.0001
FVC, L	3.4 (0.6)	3.8 (0.6)	4.0 (0.6)	4.4 (0.6)	< 0.0001	2.7 (0.5)	2.8 (0.5)	2.9 (0.5)	3.1 (0.5)	< 0.0001
FEV1, L	2.7 (0.6)	3.0 (0.6)	3.2 (0.6)	3.6 (0.6)	< 0.0001	2.2 (0.5)	2.3 (0.4)	2.4 (0.4)	2.5 (0.5)	< 0.0001
FEV1/FVC, %	78.5 (7.5)	78.6 (7.6)	79.8 (6.4)	80.8 (5.7)	< 0.0001	81.4 (6.2)	81.7 (5.9)	81.6 (5.7)	82.2 (5.8)	0.0018
Blood pressure										
SBP, mmHg	138.6 (18.2)	135.3 (16.7)	134.1 (16.9)	130.6 (15.0)	< 0.0001	127.4 (18.7)	127.2 (18.5)	125.1 (18.3)	122.3 (17.9)	< 0.0001
DBP, mmHg	80.7 (11.1)	81.8 (10.9)	83.3 (11.1)	82.4 (11.0)	0.0003	77.0 (10.6)	77.1 (10.6)	77.0 (10.8)	76.3 (11.1)	0.0740
Pulse rate, beat/min	65.8 (11.2)	64.0 (10.1)	65.4 (10.2)	65.3 (9.9)	0.0064	67.5 (9.5)	66.7 (9.3)	66.6 (9.4)	66.4 (9.5)	0.0047
CIMT, mm	0.7 (0.2)	0.7 (0.1)	0.6 (0.1)	0.6 (0.1)	< 0.0001	0.6 (0.1)	0.6 (0.1)	0.6 (0.1)	0.5 (0.1)	< 0.0001
Lipid markers										
TC, mg/dL	199.5 (32.5)	203.9 (33.6)	206.8 (36.8)	205.0 (36.0)	**0.0007**	211.4 (35.3)	213.3 (35.9)	214.8 (35.9)	209.6 (36.5)	0.0004
Triglycerides, mg/dL	117.3 (81.0)	123.2 (91.6)	128.3 (116.7)	135.9 (116.5)	0.0027	95.0 (55.6)	95.2 (59.2)	92.1 (83.4)	86.7 (56.6)	0.0002
HDL-C, mg/dL	57.6 (15.3)	57.7 (15.5)	56.9 (14.1)	56.7 (15.0)	0.4408	66.3 (15.9)	67.4 (15.6)	69.0 (16.1)	68.8 (16.5)	< 0.0001
Glucometabolic markers										
HbA1c, %	5.6 (0.5)	5.5 (0.5)	5.5 (0.5)	5.4 (0.4)	< 0.0001	5.4 (0.4)	5.4 (0.4)	5.4 (0.4)	5.4 (0.3)	< 0.0001
Glucose, mg/dL	92.9 (18.5)	90.6 (14.7)	89.9 (16.0)	87.8 (12.7)	< 0.0001	87.3 (14.0)	85.7 (12.2)	85.2 (11.3)	84.0 (10.2)	< 0.0001
eGFR, ml/min/1.73 m^2^	90 (21.3)	95.6 (19.6)	102.6 (19.7)	110.8 (18.3)	< 0.0001	103.9 (24.0)	106.7 (22.4)	108.6 (21.9)	114.3 (22.2)	< 0.0001
Uric acid, mg/dL	5.7 (1.3)	5.9 (1.2)	6 (1.2)	6.1 (1.2)	< 0.0001	4.4 (1.0)	4.5 (1.0)	4.5 (1.0)	4.4 (1.0)	0.3058
Red blood cells count, 10^4^/µL	468.1 (43.1)	475.7 (38.6)	482.8 (38.3)	491.4 (37.6)	< 0.0001	440.3 (33.2)	441.6 (33.0)	442.9 (31.6)	443.7 (31.7)	0.0095
Hemoglobin, g/dL	14.7 (1.3)	14.9 (1.1)	15.1 (1.1)	15.2 (1.0)	< 0.0001	13.2 (1.1)	13.2 (1.1)	13.3 (1.0)	13.2 (1.1)	0.6817
Hematocrit, %	44.2 (3.5)	44.8 (3.0)	45.3 (3.0)	45.5 (2.8)	< 0.0001	40.6 (3)	40.6 (3.0)	40.8 (2.8)	40.6 (2.9)	0.3917

Data are expressed as mean (standard deviation) for continuous variables and number (percentage) for categorical variables

GS: grip strength; BMI: body mass index; WC: waist circumference; %BFM; percentage body fat mass VC: vital capacity; FVC: forced vital capacity; FEV1: forced expiratory volume in 1 s; SBP: systolic blood pressure; DBP: diastolic blood pressure; TC: total cholesterol; CIMT: carotid intima-media thickness; HDL-C: high-density lipoprotein cholesterol; HbA1c: hemoglobin A1c; and eGFR: estimated glomerular filtration rate; Q: quartile

The p-value was obtained using ANOVA for continuous variables and chi-square for categorical variables of proportion

After adjusting for age, body composition, and smoking status, leg extension strength was positively associated with bone area ratio, VC, FVC, FEV1, total cholesterol, eGFR, and uric acid, and negatively associated with WC, percentage body fat mass, and pulse rate in both the sexes (Table 4). In men, leg extension strength was positively associated with diastolic blood pressure. In women, it was positively associated with HDL cholesterol, HbA1c, red blood cell count, hemoglobin, and hematocrit, and negatively associated with glucose.

**Table 4:** Association between leg extension strength and physiological data adjusted for age, body composition, and smoking

	Men			Women		
	N	β	p-value	N	β	p-value
Anthropometry						
WC^a^, cm	2855	–9.733	< 0.0001	6701	–2.484	< 0.0001
%BFM, %	2851	–12.951	< 0.0001	6704	–3.846	< 0.0001
Bone area ratio, %	2858	4.681	< 0.0001	6713	1.572	< 0.0001
Pulmonary function						
VC^b^, L	2716	71.791	< 0.0001	6375	43.397	< 0.0001
FVC^b^, L	2708	57.465	< 0.0001	6350	37.576	< 0.0001
FEV1^b^, L	2708	50.820	< 0.0001	6350	38.616	< 0.0001
FEV1/FVC^b^, %	2708	–0.801	0.0957	6350	–0.256	0.2473
Blood pressure						
SBP, mmHg	2857	–0.069	0.7291	6705	–0.775	0.3084
DBP, mmHg	2857	0.895	0.0019	6705	0.122	0.2940
Pulse rate, beat/min	2857	–1.028	0.0007	6705	–0.461	0.0003
CIMT, mm	2845	–18.792	0.4605	6677	5.352	0.6621
Lipid markers						
TC, mg/dL	2858	0.332	0.0002	6710	0.175	< 0.0001
Triglycerides, mg/dL	2858	0.042	0.1840	6710	–0.023	0.2465
HDL-C, mg/dL	2858	0.402	0.0684	6710	0.409	< 0.0001
Glucometabolic markers						
HbA1c, %	2856	–6.626	0.3348	6701	8.550	0.0110
Glucose, mg/dL	2856	–0.315	0.1233	6701	–0.417	< 0.0001
eGFR, ml/min/1.73 m^2^	2858	0.870	< 0.0001	6710	0.214	0.0043
Uric acid, mg/dL	2858	5.641	0.0308	6709	2.879	0.0260
Red blood cells count, 10^4^/µL	2858	0.020	0.8124	6708	0.097	0.0099
Hemoglobin, g/dL	2858	3.066	0.2846	6708	3.529	0.0017
Hematocrit, %	2858	0.786	0.4581	6708	1.131	0.0072

The general linear model was adjusted for age, smoking status, and body mass index

^a^Adjusted for weight instead of body mass index

^b^Adjusted for height instead of body mass index

WC: waist circumference; %BFM: percentage body fat mass; VC: vital capacity; FVC: forced vital capacity; FEV1: forced expiratory volume in 1 s; SBP: systolic blood pressure; DBP: diastolic blood pressure; CIMT: carotid intima-media thickness; TC: total cholesterol; HDL-C: high-density lipoprotein cholesterol; HbA1c: hemoglobin A1c; and eGFR: estimated glomerular filtration rate

In the subgroup analysis according to age (< 65, 65–74, > 74 years), pulse rate for < 65-year-old men, CIMT for < 65-year-old women, triglycerides for > 74-year-old men, hemoglobin for > 74-year-old women, and hematocrit for > 74-year-old women showed a new significant association with grip strength; and FEV1/FVC for < 65-year-old men and HbA1c for 65–74-year-old men showed a new significant association with leg extension strength. Although several physiological factors were no longer significantly associated with grip or leg extension strength, no major changes were observed in the associations between physiological data and grip or leg extension strength (Supplementary Table 1, Supplementary Table 2).

For clarity regarding the associations between the physiological data and muscle strength, Table 5 summarizes the results of Tables 2 and 4. Furthermore, Table 6 summarizes the differences in the association of physiological data between grip and leg extension strength.

**Table 5:** Summary of Tables 2 and 4

	Men		Women	
	GS	LS	GS	LS
Anthropometry				
WC^a^, cm	↓	↓	↓	↓
%BFM, %	↓	↓	↓	↓
Bone area ratio, %	↑	↑	↑	↑
Pulmonary function				
VC^b^, L	↑	↑	↑	↑
FVC^b^, L	↑	↑	↑	↑
FEV1^b^, L	↑	↑	↑	↑
FEV1/FVC^b^, %	-	-	-	-
Blood pressure				
SBP, mmHg	-	-	↑	-
DBP, mmHg	↑	↑	↑	-
Pulse rate, beat/min	-	↓	-	↓
CIMT, mm	↓	-	-	-
Lipid markers				
TC, mg/dL	-	↑	-	↑
Triglycerides, mg/dL	-	-	-	-
HDL-C, mg/dL	-	-	↑	↑
Glucometabolic markers				
HbA1c, %	↓	-	↓	↑
Glucose, mg/dL	-	-	-	↓
eGFR, ml/min/1.73 m^2^	↑	↑	↑	↑
Uric acid, mg/dL	-	↑	-	↑
Red blood cell count, 10^4^/µL	-	-	↑	↑
Hemoglobin, g/dL	-	-	-	↑
Hematocrit, %	-	-	-	↑

The general linear model was adjusted for age, smoking status, and body mass index

^a^Adjusted for weight instead of body mass index

^b^Adjusted for height instead of body mass index

WC: waist circumference; %BFM: percentage body fat mass; GS: grip strength; LS: leg extension strength; WC: waist circumference; %BFM: percentage body fat mass; VC: vital capacity; FVC: forced vital capacity; FEV1: forced expiratory volume in 1 s; SBP: systolic blood pressure; DBP: diastolic blood pressure; CIMT: carotid intima-media thickness; TC: total cholesterol; HDL-C: high-density lipoprotein cholesterol; HbA1c: hemoglobin A1c; and eGFR: estimated glomerular filtration rate; ↑: *p* < 0.05 for positive association; ↓: *p* < 0.05 for negative association; -: not significant associated

**Table 6:** Summary of differences in the association of physiological data between grip and leg extension strength

**Consistent positive association in both the sexes**
Bone area ratio, VC, FVC, FEV1, eGFR
**Consistent negative association in both the sexes**
WC, %BFM
**Consistent positive association in men**
DBP
**Consistent positive association in women**
HDL-C, Red blood cell count
**Inconsistent association in both the sexes**
Pulse rate, TC, HbA1c, Uric acid
**Inconsistent association in men**
CIMT
**Inconsistent association in women**
SBP, DBP, Glucose, Hemoglobin, Hematocrit

WC: waist circumference; %BFM: percentage body fat mass; VC: vital capacity; FVC: forced vital capacity; FEV1: forced expiratory volume in 1 s; SBP: systolic blood pressure; DBP: diastolic blood pressure; CIMT: carotid intima-media thickness; TC: total cholesterol; HDL-C: high-density lipoprotein cholesterol; HbA1c: hemoglobin A1c; and eGFR: estimated glomerular filtration rate

## Discussion

Through a large community-based cohort, we evaluated the association between physiological data and muscle strength (i.e., grip and leg extension strength). Grip and leg extension strength were positively associated with bone area ratio, VC, FVC, FEV1, and eGFR and negatively associated with the WC and percentage body fat mass in both the sexes. Diastolic blood pressure was positively associated with grip strength in both the sexes and leg extension strength in men, but not women. In women, higher grip and leg extension strength was associated with higher HDL cholesterol and red blood cell counts. Nonetheless, these associations were not confirmed in men. Total cholesterol and uric acid levels were positively associated with leg extension strength, but not grip strength. Furthermore, in women, we observed differences in the association between HbA1c and grip and leg extension strength. In summary, the association between grip strength and physiological data was mostly consistent with that between leg extension strength and physiological data. Several physiological parameters, such as pulse rate, total cholesterol, uric acid, and HbA1c, were inconsistent with the association between grip and leg extension strength.

### Average muscle strength according to the age group

Grip and leg extension strength declined markedly from 50 years of age. Furthermore, the decline in muscle strength differed according to sex. Abe et al. reported that grip strength declined after the age of 50 years in 613 Japanese adults aged 20–89 years [43]. Makizako et al. studied 10,092 community-dwelling Japanese older adults and found that the patterns of age-dependent decrease in physical performance measures, including grip strength, also differed among the performance measures and between the sexes [44]. Our results are consistent with those of previous studies. Beside a decrease in muscle mass, which is a major factor in the age-related decline in muscle strength, physical inactivity is also an important factor [45]. In this study, the step counts were maintained from age 70 to 79 years, except after the age of 80 years. Therefore, the decline in muscle strength in the age group of the participants in the present study may be primarily due to age-related loss of muscle mass or a decrease in the intensity of exercise to maintain muscle strength, rather than physical inactivity.

### Association between physiological data and muscle strength

Grip and leg extension strength demonstrated similar associations with WC, percentage body fat mass, bone area ratio, pulmonary function, and eGFR in both the sexes. These associations were consistent with those reported in previous cross-sectional studies on anthropometry [9, 46], pulmonary function [17, 47], and eGFR [41]. Thus, muscle strength assessed using grip or leg extension strength may be a useful marker of these functions.

HDL cholesterol and red blood cell count were associated with grip and leg extension strength only in women. In men, neither grip nor leg extension strength was significantly associated, but the regression coefficient for HDL cholesterol was greater in men than in women. We thought that the smaller sample size or larger standard deviation in men accounted for this lack of statistical significance. However, the standard deviation was not larger in men. Thus, we concluded that our sample size was not large enough to detect such a small difference in men.

CIMT was negatively associated with grip strength in men. Conversely, Yamanashi et al. investigated the association between grip strength and CIMT in 3,136 Japanese participants (1,234 men and 1,902 women) aged > 40 years and reported no significant association [14]. Our study included younger participants and a larger sample size, which may explain this discrepancy.

Diastolic blood pressure was positively associated with grip and leg extension strength in men. In women, systolic and diastolic blood pressures were positively associated with grip strength. Blanchard et al. reported that upper- and lower-body muscle strengths were greater among adults with pre-established hypertension than adults with normal blood pressure [48]. In contrast, Gubelmann et al. investigated the association between grip strength and cardiovascular risk markers in 3,468 Swiss participants (1,577 men and 1,891 women) aged 50–75 years and reported no significant association between grip strength and blood pressure levels [9]. Notably, the large sample size in our study may have facilitated detecting the relatively small associations to be detected in sample sizes used previously [9]. The positive association between blood pressure and grip strength can be explained as follows: Takase et al. reported that the fat-free mass index was positively associated with the prevalence of hypertension [20]. Markus et al. reported that lower grip strength was associated with lower structural and functional parameters of the heart [49]. Thus, high muscle mass and grip strength may serve as markers of cardiac strength and high blood pressure, respectively.

Pulse rate was negatively associated with leg extension strength in both the sexes. Our grip strength results are consistent with those of previous cross-sectional studies that demonstrated no significant association between grip strength and heart rate [17, 49]. We could not identify reports indicating an association between leg strength and pulse rate; nonetheless, exercises that maintain and improve the lower extremity muscle strength decreases the resting heart rate [50], thus supporting our results.

Total cholesterol level was positively associated with leg extension strength in both the sexes. Kohl reported that lower-body leg press strength was not significantly associated with total cholesterol in a group of 6,653 American participants aged 20–69 years (5,460 men and 1,193 women) [51]. Our results, which did not demonstrate significant associations between grip strength and total cholesterol in either sex, are partly consistent with those of a previous cross-sectional study that indicated a positive association between the grip strength and total cholesterol in men, but no significant association in women [9]. As our study included Japanese participants aged ≥ 20 years, the differences in participant characteristics may have affected our results.

Uric acid level was positively associated with leg extension strength in both the sexes. Similarly, peak isokinetic knee extensor strength was positively associated with uric acid level in both the sexes [52]. Researchers have mentioned the lack of a unified view on the association between uric level and grip strength, with reports suggesting a positive association in women [53] or no significant association [9].

Hemoglobin and hematocrit levels were positively associated with leg extension strength in women. While there are no studies on leg extension strength in relation to hemoglobin, the following studies have reported the association between these physiological data and other lower extremity muscle strengths. Regarding lower extremity muscle strength, the hemoglobin level was not significantly associated with ankle extension [54] or knee joint strength [55]. Our results, which indicated no significant association between grip strength and these physiological parameters, are inconsistent with those of a previous cross-sectional study that demonstrated an association between grip strength and anemia [12].

HbA1c levels were negatively associated with grip strength in women. Mainous et al. studied 1,463 adults aged ≥ 20 years and mentioned that the grip strength was lower in individuals with an HbA1c level ≥ 6.5% compared with that of individuals with an HbA1c level < 6.5% [56]. Our grip strength results are consistent with those of a previous study. By contrast, leg extension strength was positively associated with HbA1c levels in women. Furthermore, a meta-analysis reported that resistance training interventions, such as increasing leg extension strength, reduced HbA1c levels in adults with type 2 diabetes mellitus [57]. The subgroup analysis according to age (Supplementary Table 2) showed that leg extension strength was positively associated with HbA1c for < 65-year-old women. In contrast, leg extension strength was negatively associated with HbA1c for 65–74-year-old men. Therefore, an inconsistent association between leg extension strength and HbA1c may be explained by differences in age structure. Additionally, in the cross-sectional analysis (Table 3), despite different ages, HbA1c levels in the higher leg extension strength group were similar to those in the lower leg extension strength group. Thus, a mechanism may increase HbA1c levels in people with lower leg extension strength. However, further studies are required delineate this mechanism.

### Strengths and limitations of the study

The strength of this study is that it is one of the largest cross-sectional studies to report the association between muscle strength and various physiological parameters in the lower and upper limbs. Furthermore, we observed the characteristics of participants with low muscle strength in relation to grip and leg extension strength. However, this study had a limitation. The cross-sectional design made it impossible to assess the causal effect of muscle strength on physiological data. Therefore, studies should include a continued follow-up of the participants to evaluate the effects of muscle strength on physiological data.

## Conclusion

This population-based cross-sectional study indicated an association between muscle strength and anthropometric and physiological data. The grip and leg extension strength of both the sexes demonstrated similar associations with anthropometry, pulmonary function, and eGFR. Furthermore, diastolic blood pressure demonstrated a similar association with grip and leg extension strength in men. HDL cholesterol and red blood cell counts indicated similar associations with grip and leg extension strength in women. Grip and leg extension strength displayed variable associations with HbA1c levels in women. The impact of muscle strength on physiological data may partially follow different paths for grip and leg extension strength. Further longitudinal studies are required to determine the causal effect of muscle strength on physiological data.

### Electronic supplementary material

Below is the link to the electronic supplementary material.

Supplementary Material 1: Supplementary Table 1. Grip strength and physiological data by age groups. Supplementary Table 2. Leg extension strength and physiological data by age groups.

## Data Availability

All data used to support the findings of this study shall be released upon application to the Tohoku Medical Megabank Organization (Sendai, Japan), which can be contacted through Prof. Atsushi Hozawa (email: hozawa@megabank.tohoku.ac.jp).

## Abbreviations

ANOVA: Analysis of Variance
BMI: Body Mass Index
CIMT: Carotid Intima-Media Thickness
eGFR: Estimated Glomerular Filtration Rate
FEV1: Forced Expiratory Volume in One Second
FVC: Forced Vital Capacity
HDL: High-Density Lipoprotein
HbA1c: Hemoglobin A1c
IMT: Intima-Media Thickness
VC: Vital Capacity
WC: Waist Circumference
