# Supplementary material for: Association of physiological factors with grip and leg extension strength: tohoku medical megabank community-based cohort study
Source: BMC Public Health. 2024 Mar 5;24:714. doi: 10.1186/s12889-024-18244-z (PMC10916074; doi:10.1186/s12889-024-18244-z)
Supplement: Supplementary file 1 — Supplementary Material 1: Supplementary Table 1. Grip strength and physiological data by age groups. Supplementary Table 2. Leg extension strength and physiological data by age groups. [file 12889_2024_18244_MOESM1_ESM.pdf]

Supplementary Table 1. Grip strength and physiological data by age groups

		<65 years			65–74 years			>74 years		
	SEX	N	β	p-value	N	β	p-value	N	β	p-value
Anthropometry										
WC[a], cm	M	1718	–0.446	<0.0001	899	–0.293	<0.0001	238	–0.313	<0.0001
	W	5012	–0.161	<0.0001	1444	–0.112	<0.0001	245	–0.159	0.0002
BFM, %	M	1715	–0.886	<0.0001	897	–0.501	<0.0001	239	–0.504	<0.0001
	W	5017	–0.402	<0.0001	1444	–0.327	<0.0001	243	–0.375	<0.0001
Bone area ratio, %	M	1720	0.169	0.0003	900	0.041	0.4651	238	0.062	0.5331
	W	5021	0.101	<0.0001	1448	0.032	0.3565	244	0.100	0.2193
Pulmonary function										
VC[b], L	M	1633	2.935	<0.0001	861	1.705	<0.0001	222	2.612	0.0003
	W	4759	2.608	<0.0001	1389	2.014	<0.0001	227	1.891	0.0119
FVC[b], L	M	1627	2.699	<0.0001	860	1.473	0.0002	221	1.788	0.0191
	W	4740	2.626	<0.0001	1383	1.853	<0.0001	227	1.234	0.1121
FEV1[b], L	M	1627	3.004	<0.0001	860	1.737	0.0001	221	2.221	0.0060
	W	4740	2.807	<0.0001	1383	2.053	<0.0001	227	1.946	0.0323
FEV1/FVC[b], %	M	1627	–0.004	0.8923	860	0.029	0.2948	221	0.073	0.1067
	W	4740	–0.001	0.9141	1383	–0.006	0.7675	227	0.059	0.1262
Blood pressure										
SBP, mmHg	M	1720	0.006	0.6488	901	0.009	0.4034	236	0.030	0.1347
	W	5016	0.010	0.0150	1446	0.018	0.0021	243	–0.002	0.8781
DBP, mmHg	M	1720	0.000	0.9914	901	0.058	0.0014	236	0.037	0.2804

	W	5016	0.005	0.4137	1446	0.030	0.0039	243	–0.018	0.4377
Pulse rate, beat/min	M	1720	–0.042	0.0170	901	0.013	0.4637	236	–0.052	0.0905
	W	5016	–0.001	0.8922	1446	–0.006	0.5259	243	–0.011	0.7141
CIMT, mm	M	1713	–1.280	0.4230	895	–2.357	0.0967	237	–1.241	0.5966
	W	4995	1.810	0.0122	1438	–0.095	0.9075	244	1.863	0.3782
Lipid markers										
TC, mg/dL	M	1719	–0.005	0.2715	900	0.000	0.9645	239	0.003	0.7953
	W	5018	–0.003	0.1307	1447	0.003	0.3086	245	–0.012	0.1335
Triglycerides, mg/dL	M	1719	0.000	0.9277	900	–0.001	0.8046	239	–0.018	0.0412
	W	5018	–0.002	0.1112	1447	–0.003	0.0901	245	–0.006	0.1701
HDL-C, mg/dL	M	1719	0.019	0.1401	900	–0.009	0.4821	239	0.037	0.1723
	W	5018	0.007	0.0915	1447	0.001	0.9207	245	–0.018	0.3014
Glucometabolic markers										
HbA1c, %	M	1718	–0.529	0.1730	900	–1.286	0.0022	238	–1.068	0.1831
	W	5009	–0.374	0.0467	1447	–0.652	0.0138	245	–0.019	0.9655
Glucose, mg/dL	M	1718	–0.006	0.6259	900	–0.008	0.5284	238	–0.016	0.4127
	W	5009	0.002	0.7364	1447	–0.006	0.4804	245	–0.001	0.3899
eGFR, ml/min/1.73 m^2	M	1719	0.039	0.0006	900	0.018	0.1458	239	0.066	0.0064
	W	5018	0.011	0.0036	1447	–0.007	0.3820	245	0.016	0.3702
Uric acid, mg/dL	M	1719	0.238	0.1061	900	–0.068	0.6784	239	–0.006	0.9847
	W	5017	–0.121	0.0772	1447	–0.081	0.4467	245	0.364	0.1155
Red blood cell count, 10^4/μL	M	1719	–0.003	0.5166	900	0.004	0.4057	239	–0.003	0.7069
	W	5016	0.002	0.3113	1447	–0.001	0.8198	245	0.020	0.0051
Hemoglobin, g/dL	M	1719	–0.114	0.5074	900	0.235	0.1605	239	–0.050	0.8647

	W	5016	−0.029	0.6102	1447	0.034	0.7507	245	0.554	0.0273
Hematocrit, %	M	1719	−0.020	0.7573	900	0.082	0.1799	239	0.036	0.7333
	W	5016	−0.003	0.8821	1447	0.018	0.6389	245	0.233	0.0083

The general linear model was adjusted for age, smoking status, and body mass index.

a: adjusted for weight instead of body mass index; b: adjusted for height instead of body mass index. M: men; W: women; WC: waist circumference; %BFM: percentage body fat mass; GS: grip strength; LS: leg extension strength; WC: waist circumference; %BFM: percentage body fat mass; VC: vital capacity; FVC: forced vital capacity; FEV1: forced expiratory volume in 1 s; SBP: systolic blood pressure; DBP: diastolic blood pressure; CIMT: carotid intima-media thickness; TC: total cholesterol; HDL-C: high-density lipoprotein cholesterol; HbA1c: hemoglobin A1c; and eGFR: estimated glomerular filtration rate.

Supplementary Table 2. Leg extension strength and physiological data by age groups

		<65 years			65–74 years			>74 years		
	SEX	N	β	p-value	N	β	p-value	N	β	p-value
Anthropometry										
WC[a], cm	M	1718	–11.297	<0.0001	899	–7.294	<0.0001	238	–4.484	0.0142
	W	5012	–2.411	<0.0001	1444	–1.906	<0.0001	245	–1.463	0.0768
BFM, %	M	1715	–15.246	<0.0001	897	–8.735	<0.0001	239	–7.182	0.0002
	W	5017	–3.653	<0.0001	1444	–3.667	<0.0001	243	–5.324	<0.0001
Bone area ratio, %	M	1720	6.443	<0.0001	900	2.484	0.0620	238	0.433	0.8464
	W	5021	1.545	<0.0001	1448	1.816	0.0115	244	1.543	0.3138
Pulmonary function										
VC[b], L	M	1633	81.498	<0.0001	861	50.467	<0.0001	222	51.163	0.0021
	W	4759	47.833	<0.0001	1389	22.022	<0.0001	227	28.319	0.0375
FVC[b], L	M	1627	66.769	<0.0001	860	36.831	<0.0001	221	40.758	0.0197
	W	4740	42.604	<0.0001	1383	15.294	0.0132	227	5.960	0.6719
FEV1[b], L	M	1627	58.017	<0.0001	860	33.063	0.0017	221	34.721	0.0621
	W	4740	45.240	<0.0001	1383	15.397	0.0350	227	–8.094	0.6240
FEV1/FVC[b], %	M	1627	–1.723	0.0221	860	–0.491	0.4526	221	0.209	0.8402
	W	4740	–0.151	0.5777	1383	–0.210	0.5957	227	–1.080	0.1222
Blood pressure										
SBP, mmHg	M	1720	–0.003	0.9914	901	–0.376	0.1382	236	0.533	0.2536
	W	5016	–0.172	0.0714	1446	0.127	0.3001	243	–0.323	0.2145
DBP, mmHg	M	1720	0.041	0.9228	901	0.448	0.2894	236	2.262	0.0034

	W	5016	−0.122	0.3892	1446	−0.039	0.8537	243	0.185	0.6632
Pulse rate, beat/min	M	1720	−1.294	0.0035	901	−0.722	0.0862	236	−0.934	0.1811
	W	5016	−0.454	0.0035	1446	−0.274	0.1982	243	−0.432	0.4151
CIMT, mm	M	1713	−15.185	0.7061	895	−0.283	0.9932	237	9.964	0.8502
	W	4995	23.305	0.1634	1438	5.664	0.7371	244	10.070	0.7977
Lipid markers										
TC, mg/dL	M	1719	0.278	0.0255	900	0.084	0.5376	239	0.283	0.2792
	W	5018	0.055	0.2175	1447	0.145	0.0209	245	0.023	0.8778
Triglycerides, mg/dL	M	1719	0.052	0.1860	900	−0.865	0.1305	239	−0.386	0.0511
	W	5018	−0.026	0.2663	1447	−0.051	0.1598	245	−0.003	0.9715
HDL-C, mg/dL	M	1719	0.401	0.2033	900	0.068	0.8270	239	0.941	0.1240
	W	5018	0.312	0.0012	1447	0.294	0.0436	245	0.061	0.0546
Glucometabolic markers										
HbA1c, %	M	1718	2.431	0.8032	900	−25.802	0.0087	238	−10.678	0.5570
	W	5009	10.908	0.0122	1447	5.644	0.3027	245	−6.767	0.4051
Glucose, mg/dL	M	1718	−0.251	0.4022	900	−0.568	0.0535	238	−0.307	0.4885
	W	5009	−0.511	0.0002	1447	−0.275	0.1007	245	−0.260	0.2357
eGFR, ml/min/1.73 m^2	M	1719	0.686	0.0162	900	0.595	0.0353	239	1.407	0.0095
	W	5018	0.178	0.0393	1447	0.321	0.0432	245	0.346	0.2994
Uric acid, mg/dL	M	1719	8.932	0.0158	900	1.912	0.6178	239	−2.906	0.6538
	W	5017	2.096	0.1883	1447	5.575	0.0115	245	4.274	0.3208
Red blood cell count, 10^4/μL	M	1719	−0.016	0.9015	900	−0.020	0.8631	239	0.047	0.8225
	W	5016	0.021	0.6533	1447	0.162	0.0110	245	0.059	0.6640
Hemoglobin, g/dL	M	1719	1.761	0.6830	900	2.803	0.4732	239	2.721	0.6810

	W	5016	1.784	0.1774	1447	4.310	0.0541	245	5.646	0.2272
Hematocrit, %	M	1719	0.102	0.9495	900	0.864	0.5461	239	0.612	0.7978
	W	5016	0.392	0.4375	1447	1.402	0.0698	245	2.033	0.2172

The general linear model was adjusted for age, smoking status, and body mass index.

a: adjusted for weight instead of body mass index; b: adjusted for height instead of body mass index. M: men; W: women; WC: waist circumference; %BFM: percentage body fat mass; GS: grip strength; LS: leg extension strength; WC: waist circumference; %BFM: percentage body fat mass; VC: vital capacity; FVC: forced vital capacity; FEV1: forced expiratory volume in 1 s; SBP: systolic blood pressure; DBP: diastolic blood pressure; CIMT: carotid intima-media thickness; TC: total cholesterol; HDL-C: high-density lipoprotein cholesterol; HbA1c: hemoglobin A1c; and eGFR: estimated glomerular filtration rate.
